# The in vitro effect of progesterone on the orexin system in porcine uterine tissues during early pregnancy

**DOI:** 10.1186/s13028-018-0430-4

**Published:** 2018-11-26

**Authors:** Kamil Dobrzyn, Nina Smolinska, Marta Kiezun, Karol Szeszko, Edyta Rytelewska, Katarzyna Kisielewska, Marlena Gudelska, Tadeusz Kaminski

**Affiliations:** Department of Animal Anatomy and Physiology, Faculty of Biology and Biotechnology, University of Warmia and Mazury in Olsztyn, Oczapowskiego 1A, 10-719 Olsztyn-Kortowo, Poland

**Keywords:** Early pregnancy, Orexins, Orexin receptors, OXA, OXB, Porcine uterus, Progesterone

## Abstract

**Background:**

Orexin A (OXA) and orexin B (OXB) are hypothalamic-derived peptides that participate in the regulation of energy metabolism, food intake and reproductive function by influencing the hypothalamic-pituitary-ovarian axis. Orexins are also produced in the endometrium, myometrium and placenta, which suggests that they could act as a link between energy metabolism and the reproductive system. Changes in the expression of orexin and the orexin receptor genes and proteins during the oestrous cycle and early gestation in pigs imply that orexin activity may be regulated by local factors within the uterus. The aim of this study was to investigate the influence of progesterone (P_4_) on the expression of orexin system genes, and proteins in the porcine uterus during early gestation. Gene expression was analyzed by real-time PCR. Adiponectin secretion was determined by ELISA, and the receptors proteins content was defined using western blot analysis.

**Results:**

In the endometrium, P_4_ enhanced OXA secretion on days 10 to 11 of gestation and OXB secretion on days 12 to 13. In the myometrium, P_4_ inhibited the secretion of both orexins on days 15 to 16 and OXB secretion also on days 12 to 13. In the endometrium, P_4_ inhibited the expression of orexin receptor 1 (OX1R) protein at nearly all times analyzed, whereas the expression of orexin receptor 2 (OX2R) protein was inhibited only on days 15 to 16 of gestation. In the myometrium, P_4_ stimulated OX1R protein expression on days 12 to 13 and 15 to 16 of gestation and inhibited OX1R protein expression on days 27 to 28. The expression of OX2R protein in the myometrium increased on days 12 to 13 and decreased on days 10 to 11 and 15 to 16.

**Conclusions:**

The results indicate that P_4_ could regulate the expression of the orexin system in the porcine uterus during early pregnancy, which suggests the presence of a local feedback loop that could play an important role in the regulation of maternal metabolism during pregnancy. The findings may contribute to the existing knowledge of the mechanisms linking maternal energy metabolism with the regulation of the reproductive system during pregnancy.

## Background

It is generally acknowledged that reproductive success of animals is largely determined by their nutritional status. Orexin A (OXA) and orexin B (OXB) are hypothalamic neuropeptides which are derived by proteolytic cleavage from a common 130-amino acid precursor called prepro-orexin (PPO). OXA is a 33-amino-acid peptide with molecular mass of 3.5 kDa, whereas OXB is a 28-amino-acid peptide with molecular mass of 2.9 kDa. There is around 46% homology between OXA and OXB. Orexin activity is mediated by G-protein-coupled receptors: orexin receptor type 1 (OX1R) and orexin receptor type 2 (OX2R). OX1R is highly selective for OXA, whereas OX2R has a similar affinity for both OXA and OXB [1, 2].

Current data suggest that orexins may influence reproductive function. Orexins were found to participate in the control of the hypothalamic–pituitary–ovarian axis [3–9]. The hormones and their receptors are also expressed locally in rat and porcine ovaries [3, 7, 10]. The elements of the orexin system have been observed in feline, canine and human placenta [11, 12]. The expression of orexin system genes and proteins was also confirmed in the porcine uterus, conceptus and trophoblasts [13, 14]. Our previous research demonstrated changes in the endometrial and myometrial expression of orexin and orexin receptor genes and proteins during the oestrous cycle and early pregnancy, which indicates that orexin expression could be dependent on the local hormonal status, including the presence and concentration of steroid hormones [7, 13, 14].

Steroids are one of the most important factors in the porcine reproductive system, and they participate in the maternal recognition and maintenance of pregnancy [15]. Steroidogenesis is not limited to the ovaries and adrenals, and it could also take place in the uterus [16]. The regulation of orexin and orexin receptor expression in the uterus is poorly understood. The aim of this study was to investigate the influence of progesterone (P_4_) on the expression of prepro-orexin (*PPO*), *OX1R* and *OX2R* genes, on OXA and OXB secretion in vitro and the expression of OX1R and OX2R proteins in the porcine endometrium and myometrium on days 10 to 11, 12 to 13, 15 to 16 and 27 to 28 of pregnancy and on days 10 to 11 of the oestrous cycle.

## Methods

### Animals and tissue collection

All studies were conducted in accordance with ethical standards of the Animal Ethics Committee at the University of Warmia and Mazury in Olsztyn, Poland. Mature gilts (Large White x Polish Landrace) at the age of 7–8 months and weight of 120–130 kg descended from private breeding farm were used in the study. Twenty-five gilts were assigned to one of five experimental groups (n = 5 per group) as follows: 10 to 11 (the beginning of maternal recognition of pregnancy), 12 to 13 (the end of maternal recognition of pregnancy), 15 to 16 (implantation) and 27 to 28 (the end of implantation) days of pregnancy and days 10 to 11 of the oestrous cycle (mid-luteal phase, connected with the period of fully active corpora lutea, corresponding to the activity of corpora lutea during pregnancy). Cyclic gilts were daily observed for estrus behavior in the presence of an intact boar. The day of onset of the second estrus was marked as day 0 of the oestrous cycle. The phase of the oestrous cycle was also confirmed on the basis of morphology of the ovaries [17]. The level of serum P_4_ was determined to confirm the phase of the oestrous cycle. The results of P_4_ measure were published in another study conducted on the same animals [18]. In the case of pregnant gilts, the day after coitus was marked as the first day of pregnancy. Insemination was performed on days 1 to 2 of the oestrous cycle. Pregnancy was confirmed by the presence of conceptuses. Uteri collected after slaughter were placed in ice-cold PBS supplemented with 100 IU/mL penicillin and 100 µg/mL streptomycin and transported to the laboratory on ice within 1 h for in vitro tissue culture.

### Tissue cultures

Endometrial and myometrial explants were performed based on a modification of the technique described by Franczak [19] with the modification of Smolinska et al. [20]. The endometrial and myometrial tissues, from the middle of uterine horns were cut into small, irregular slices with about 3 mm of thickness (100 mg ± 10%). On days 27 to 28 of pregnancy, conceptuses and trophoblasts were dissected from the endometrium. All slices of the uteri on days 27 to 28 of pregnancy were collected from the implantation sites. Tissue explants were washed three times in medium M199 (Sigma-Aldrich, USA). Endometrial and myometrial slices were placed in the separate sterile culture vials with 2 mL medium 199 containing 0.1% BSA (MP Biomedicals, USA), 5% dextran/charcoal-stripped newborn calf serum (Sigma-Aldrich), penicillin (100 IU/mL) and streptomycin (100 μg/mL). Cultures were preincubated for 2 h (37 °C, 95% O_2_, 5% CO_2_). To determine the influence of P_4_ on *PPO, OX1R* and *OX2R* genes expression, OX1R and OX2R protein expression and OXA and OXB secretion, endometrial and myometrial slices were treated with P_4_ (Sigma-Aldrich) at the concentration of 10, 100 and 1000 nM and incubated for another 24 h at the same conditions. The doses of P_4_ were chosen according to Blitek et al. [21]. Control tissues were incubated without any treatment. Our preliminary studies indicated that the influence of P_4_ solvent (0.00094%, 0.0094% and 0.094% of ethanol for 10, 100 and 1000 nM of P_4_ doses, respectively) on gene/protein expression and hormone secretion was negligible. All cultures were prepared in duplicates in five separate experiments for each group (n = 5). At the end of the experiment, the media were collected and stored at − 20 °C. Endometrial and myometrial slices were frozen and stored at − 80 °C for further analyses. The viability of slices was monitored by measuring lactate dehydrogenase (LDH) activity in media after 2 h of preincubation as well as at the end of the treatment period. The release of LDH was performed using a Liquick Cor-LDH kit (Cormay, Poland) following the manufacturer’s instructions. The activity of LDH during the slices culture was compared to its activity in medium obtained after destruction of endometrial and myometrial cells by homogenization (positive control for causing cell death and the maximal release of LDH). Mean activity of LDH in cultured slices after treatment period was 55.1 ± 4.5 U/L for endometrium (1.8% of maximal release of LDH after total endometrial cells destruction) and 34.1 ± 4.9 U/L for myometrium (1.7% of maximal release of LDH after myometrial cells destruction).

### Quantitative real-time PCR

Total RNA was extracted from tissues using peqGOLD TriFast isolation system (Peqlab, Germany) according to the manufacturer’s instructions. RNA purity and quantity were measured spectrophotometricaly (Infinite M200 Pro, Tecan, Switzerland). One microgram of RNA was used in the reaction of reverse transcription in a total volume of 20 μL with 0.5 μg oligo(dT)_15_ (Roche, Switzerland). The reaction was prepared using Omniscript RT Kit (Qiagen, USA) at 37 °C for 1 h and was terminated by the incubation at 93 °C for 5 min. Quantitative real-time PCR analysis was carried out using PCR System 7300 and Power SYBR Green Master Mix (Applied Biosystems, USA), as described previously [22]. Specific primer pairs used to amplify parts of *PPO, OX1R, OX2R,* cyclophilin (*PPIA*) and β-actin (*ACTB*) genes are detailed in Table 1. The *PPIA* and *ACTB* genes, which are expressed constitutively, were used as an internal control to verify the method. During the preliminary experiments it was found that expression of *PPIA* and *ACTB* was very similar in the endometrium and myometrium and was stable during the oestrous cycle and pregnancy and with treatments. The PCR reaction mixtures contained 10 ng of cDNA, primers, 12.5 μL Power SYBR Green PCR Master Mix (Applied Biosystems), and RNase free water at the final volume of 25 μL. Real-time PCR reaction conditions for *PPO, OX1R* and *OX2R* were as follows: enzyme activation and initial denaturation at 95 °C for 10 min, afterward, 40 cycles of denaturation at 95 °C for 15 s, and annealing at 60 °C for 1 min. For *PPIA* reaction conditions were as follows: 50 °C for 2 min, then enzyme activation and initial denaturation at 95 °C for 10 min, afterward, 40 cycles of denaturation at 95 °C for 15 s, and annealing at 60 °C for 1 min. For *ACTB* reaction conditions were as follows: enzyme activation and initial denaturation at 95 °C for 10 min, followed by 40 cycles of denaturation at 95 °C for 15 s, annealing at 61 °C for 1 min and elongation at 72 °C for 1 min. Negative controls were performed in which cDNA was substituted by water, or reverse transcription was not performed before PCR. All samples were prepared in duplicates. The specificity of amplification was tested at the end of the reaction by the analysis of melting-curve. Product purity was confirmed by agarose gel electrophoresis. Calculation of the relative expression levels of *PPO, OX1R* and *OX2R* were conducted based on the comparative cycle threshold method (ΔΔCT), and normalized using the geometrical means of reference gene expression levels: *PPIA* and *ACTB*.

**Table 1:** Characteristics of primers used in the study of gene expression in porcine endometrial and myometrial explants

Gene	Primers sequences	Accession number	Complementary position	Primer, nM	References
*PPO*	F: 5′-AAGACGACACCCTTCCTGGAGAC-3′	EF434655	F: 41–63	500	[9]
	R: 5′-TGATTGCCAGCGCCGTGTAGCA-3′		R: 240–261	500	
*OX1R*	F: 5′-ACCGCTGGTATGCCATCTACCAC-3′	AF097995	F: 27–49	500	[5]
	R: 5′-ATAGAGGTCATCTGCCCAGCGTTCA-3′		R: 208–232	500	
*OX2R*	F: 5′-GACATCACGGAGACCTGGTTCTTC-3′	AF059740	F: 30–53	500	[5]
	R: 5′-GATGTAGGAGACGATCCAGATGATGAC-3′		R: 207–233	500	
*PPIA*	F: 5′-GCACTGGTGGCAAGTCCAT-3′	AY266299	F: 219–237	300	[42]
	R: 5′-AGGACCCGTATGCTTCAGGA-3′		R: 269–299	300	
*ACTB*	F: 5′-ACATCAAGGAGAAGCTCTGCTACG-3′	U07786	F: 266–289	500	[43]
	R: 5′-GAGGGGCGATGATCTTGATCTTCA-3′		R: 608–631	500	

*PPO* prepro-orexin, *OX1R* orexin type 1 receptor, *OX2R* orexin type 2 receptor, *PPIA* cyclophilin, *ACTB* β-actin; *F* forward, *R* reverse

### Enzyme-linked immunosorbent assay (ELISA) of OXA and OXB

The level of OXA and OXB protein concentrations in the culture media was determined using commercial ELISA kit (NeoBiolab, USA) according to the manufacturer’s protocol. The range of standard curves was 0.5–10 ng/mL. The sensitivity of the assays was approximately 0.1 ng/mL. The sensitivity of this assays was defined as the lowest protein concentration that could be differentiated from zero samples. Absorbance values were measured at 450 nm using Infinite M200 PRO reader with Tecan i-control software (Tecan). The data was linearized by plotting the log of OXA or OXB concentration versus the log of the optical density and the best fit line was determined by regression analysis. Intra- and interassay coefficients of variation of the OXA ELISA assay was 3.08% ± 0.97 and 3.45%, respectively. For the OXB ELISA assay intra- and interassay coefficients of variation was 4.66% ± 1.23 and 7.87%, respectively.

### Protein isolation and western blotting

The endometrial and myometrial explants, obtained from the tissue cultures were homogenized on ice with the cold Total Protein Extraction Kit lysis buffer (Genoplast, Poland) in the presence of peptidase and phosphatase inhibitors (Sigma-Aldrich, USA), and incubated on ice for 30 min. The lysates were cleared by centrifugation at 10,000×*g* for 2 × 10 min at 4 °C. The supernatants were stored at  − 80 °C for further study. The protein concentrations were determined by the Bradford dye-binding procedure with the dilutions of bovine serum albumin (BSA) as standards.

Western blotting analysis was performed as described by Smolinska et al. [23]. Equal amounts (40 μg) of endometrial and myometrial lysates were resolved by SDS-PAGE electrophoresis in 12.5% polyacrylamide gel for separating OX1R, OX2R and actin proteins and transferred onto nitrocellulose membrane (Whatman, USA). Membranes were blocked for 4 h at 4 °C in Tris-buffered saline Tween-20 containing 5% skimmed milk powder. After blocking, membranes were incubated for 12 h at 4 °C with sheep polyclonal antibodies to OX1R at a dilution of 1:100 (Abcam, UK), mouse polyclonal antibodies to OX2R at a dilution of 1:300 (Abcam, UK), or rabbit polyclonal antibodies to actin diluted 1:200 (Sigma-Aldrich, USA), which were used as a control for equal loading as well as to quantify porcine OX1R and OX2R proteins. To identify immunoreactive products, membranes were incubated for 2 h at RT with rabbit anti-sheep IgG for OX1R or goat anti-mouse IgG for OX2R (Santa Cruz Biotechnology, USA; diluted 1:500) or goat anti-rabbit IgG for actin conjugated with alkaline phosphatase (Santa Cruz Biotechnology, USA; dilution 1:2000). Nonspecific foetal calf serum (MP Biomedicals) was used instead of primary antibodies to produce negative control blots. Visualization of immunocomplexes was carried out using 4-nitroblue tetrazolium chloride (NBT) and 5-bromo-4-chloro-3-indolyl phosphate (BCIP), according to the manufacture’s protocol (Promega, USA). The results of the analysis were quantified by densitometric scanning of membranes with GelScan for Windows version 1.45 software (Kucharczyk, Poland). Data were expressed as a ratio of OX1R or OX2R protein relative to actin protein in arbitrary optical density units.

### Statistical analysis

Statistical analysis was performed using the Statistica software (StatSoft Inc., USA). All variables were analyzed using descriptive statistics (mean, standard deviation, sample minimum, and sample maximum). To determine the differences in genes expression and proteins concentration between control groups and P_4_ treated groups, one-way ANOVA followed by the Fisher’s LSD post-hoc test were used. The model included the effect of P_4_ in three different concentrations treatment. Results were reported as the mean ± SEM from five independent observations. Values for P < 0.05 were considered statistically significant.

## Results

### The effect of P_4_ on *PPO* gene expression and OXA and OXB proteins secretion by the endometrial and myometrial tissue explants

**Fig. 1:**
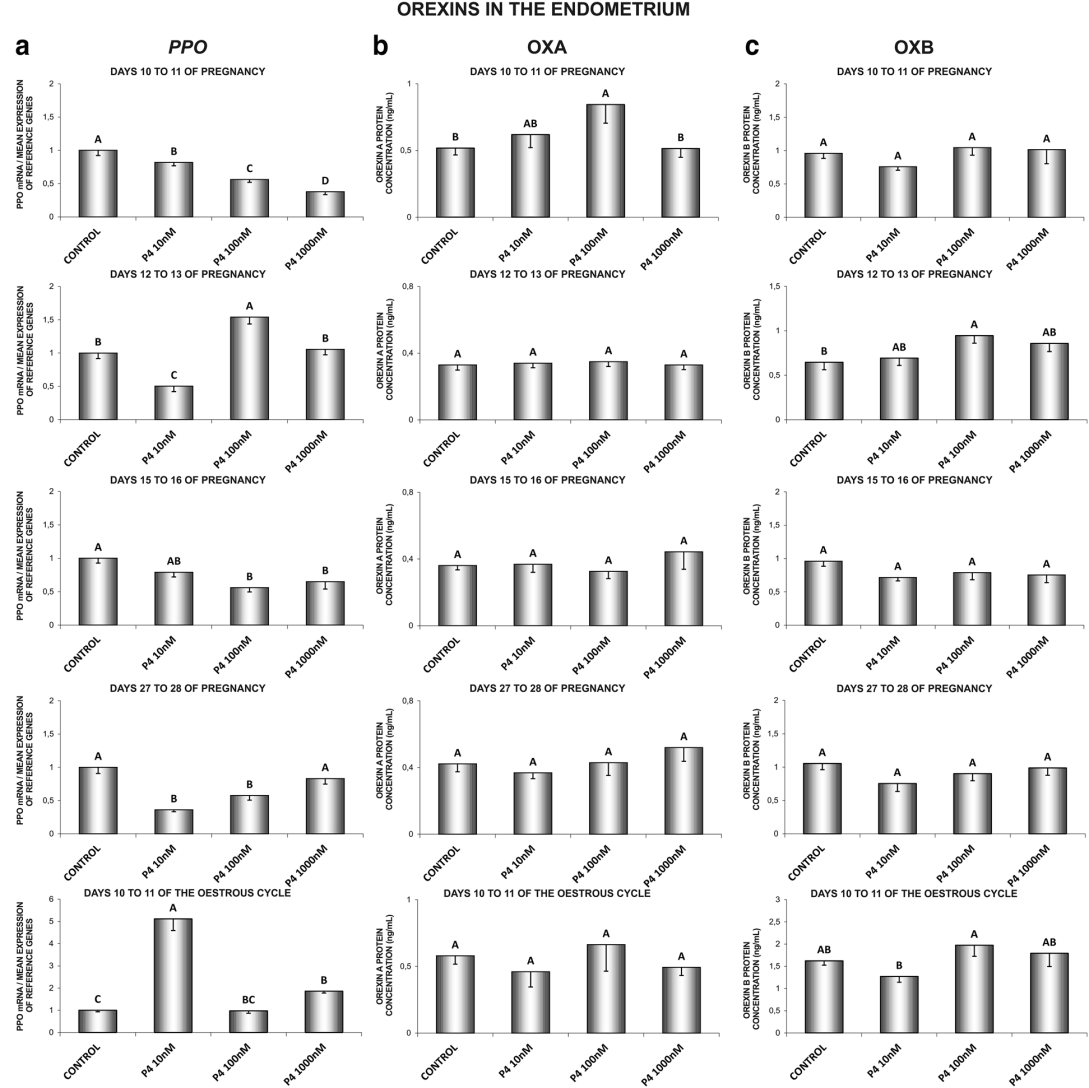
The influence of P4 (10, 100, 1000 nM) on prepro-orexin (*PPO*) mRNA expression (**a**), orexin A (OXA) (**b**) and orexin B (OXB) (**c**) proteins secretion in the porcine endometrium on days 10 to 11, 12 to 13, 15 to 16 and 27 to 28 of pregnancy, and on days 10 to 11 of the oestrous cycle. The gene expression was determined by quantitative real-time (**a**). The proteins secretion was determined by ELISA test (**b**; **c**). Results are reported as the mean ± SEM (n = 5). Bars with different superscripts differ (P < 0.05)

In the endometrial tissue explants, on days 10 to 11 of pregnancy, P_4_ decreased *PPO* gene expression (10, 100 and 1000 nM) and increased OXA (100 nM) secretion (Fig. 1a, b). On days 12 to 13 of pregnancy, P_4_ at the dose of 10 nM decreased, whereas at the dose of 100 nM increased *PPO* gene expression. On those days, P_4_ (100 nM) stimulated endometrial OXB secretion (Fig. 1a, c). On days 15 to 16 of pregnancy, P_4_ (100, 1000 nM) caused a decrease in *PPO* gene expression (Fig. 1a). Similarly, on days 27 to 28 of pregnancy, P_4_ (10, 100 nM) inhibited the gene expression (Fig. 1a). On days 10 to 11 of the oestrous cycle, P_4_ (10 and 1000 nM) stimulated *PPO* gene expression (Fig. 1a) (P < 0.05).

**Fig. 2:**
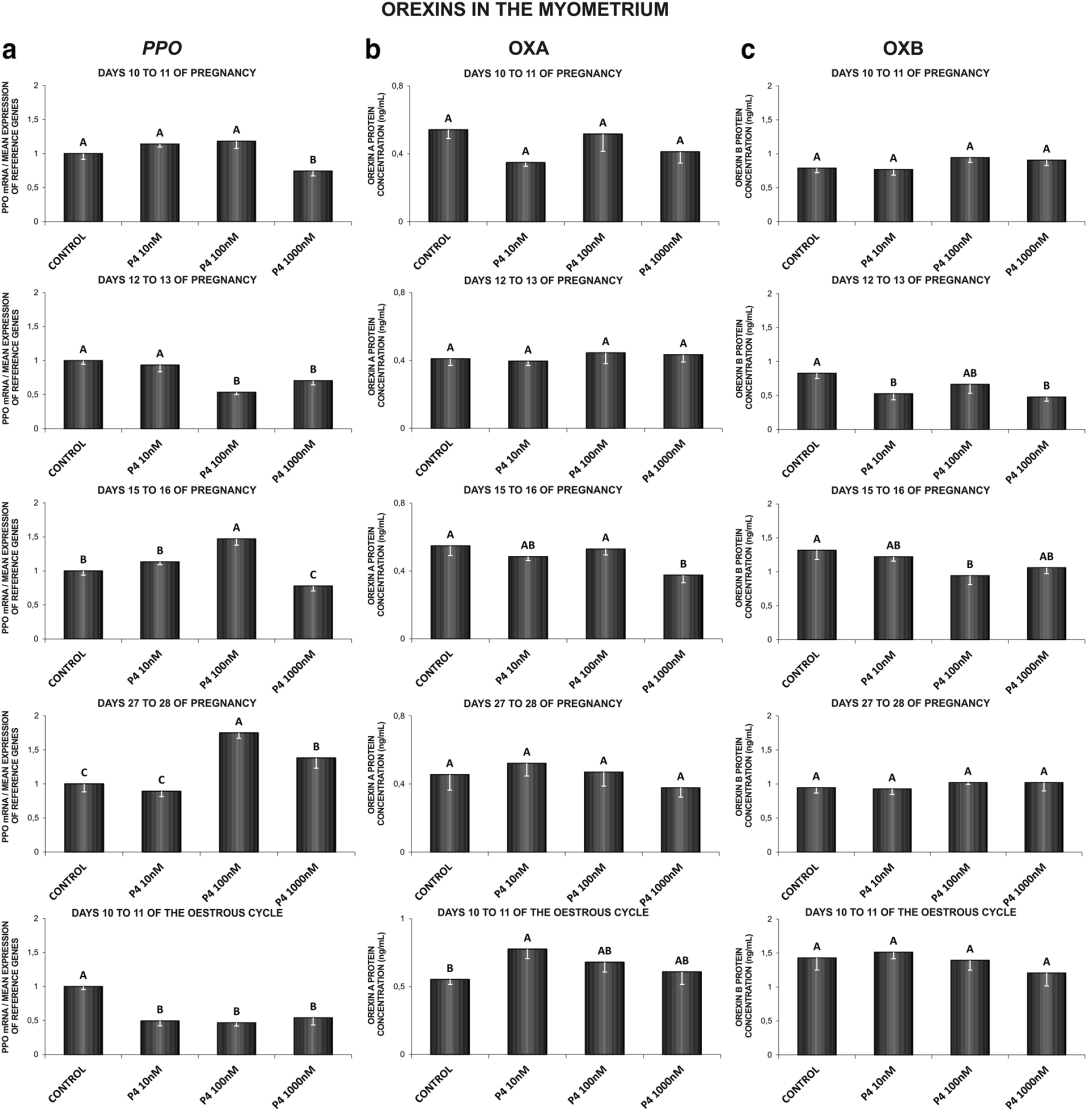
The influence of P4 (10, 100, 1000 nM) on prepro-orexin (*PPO*) mRNA expression (**a**), orexin A (OXA) (**b**) and orexin B (OXB) (**c**) proteins secretion in the porcine myometrium on days 10 to 11, 12 to 13, 15 to 16 and 27 to 28 of pregnancy, and on days 10 to 11 of the oestrous cycle. The gene expression was determined by quantitative real-time PCR (**a**). The proteins secretion was determined by ELISA test (**b**; **c**). Results are reported as the mean ± SEM (n = 5). Bars with different superscripts differ (P < 0.05)

In the myometrium, on days 10 to 11 of pregnancy, P_4_ (1000 nM) caused a decrease in *PPO* mRNA content (Fig. 2a). On days 12 to 13 of pregnancy, P_4_ inhibited *PPO* gene expression (100, 1000 nM) and OXB secretion (10, 1000 nM) (Fig. 2a, c). On days 15 to 16 of gestation, P_4_ at the dose of 100 nM stimulated, but at the dose of 1000 nM inhibited *PPO* gene expression. On those days, P_4_ inhibited the myometrial secretion of both, OXA (1000 nM) and OXB (100 nM) (Fig. 2a–c). On days 27 to 28 of gestation, P_4_ enhanced *PPO* gene expression (100, 1000 nM) (Fig. 2a). On days 10 to 11 of the oestrous cycle, P_4_ inhibited myometrial *PPO* gene expression (10, 100, 1000 nM), but increased OXA secretion (10 nM) (Fig. 2a, b) (P < 0.05).

### The effect of P_4_ on OX1R gene and protein expression in the endometrial and myometrial tissue explants

**Fig. 3:**
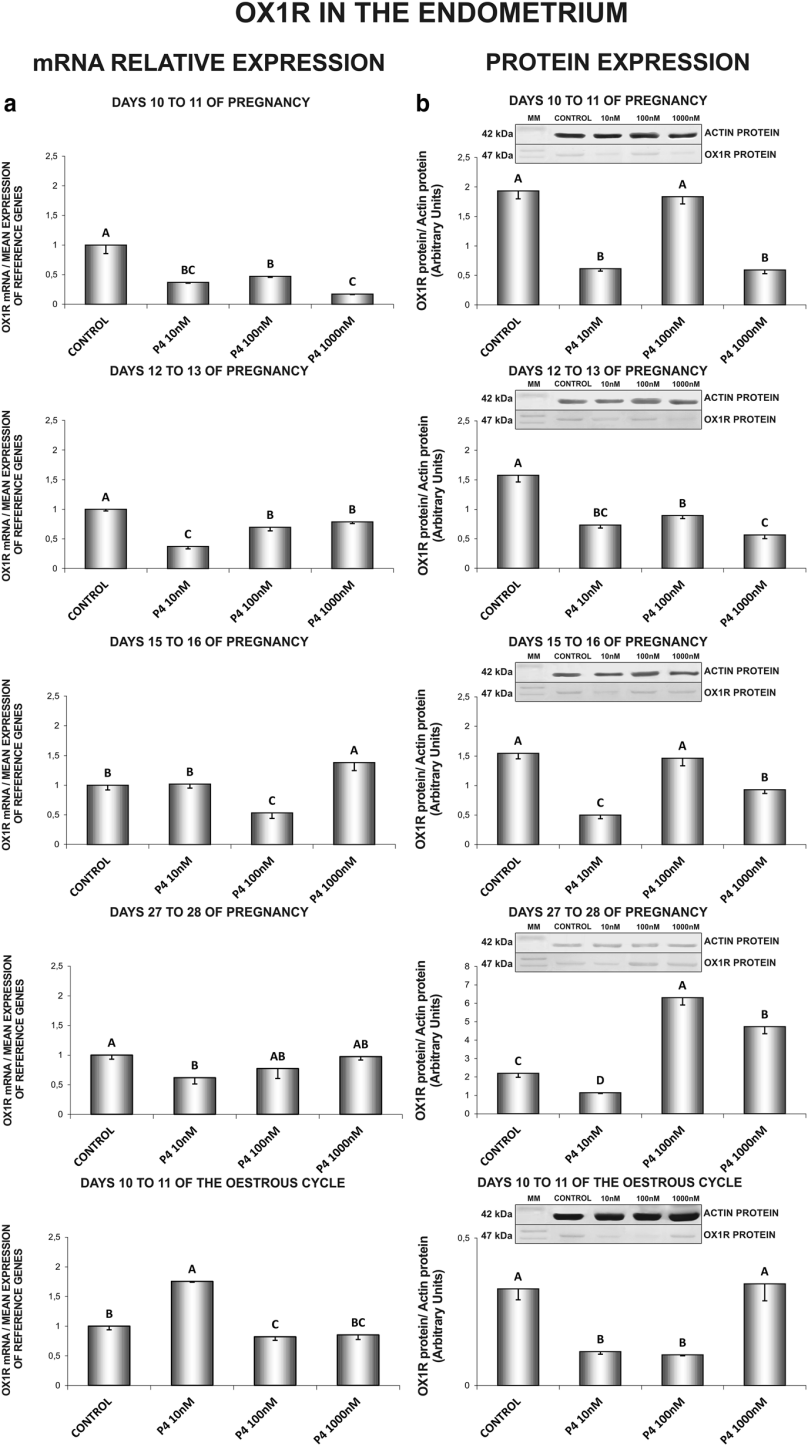
The influence of P4 (10, 100, 1000 nM) on orexin type 1 receptor (OX1R) mRNA (**a**) and protein (**b**) expression in the porcine endometrium on days 10 to 11, 12 to 13, 15 to 16 and 27 to 28 of pregnancy, and on days 10 to 11 of the oestrous cycle. The gene expression was determined by quantitative real-time PCR (**a**). The protein concentration was determined by the western blotting analysis (**b**); upper panels: representative immunoblots (MM—molecular marker); lower panels: densitometric analysis of OX1R protein relative to actin protein. Results are reported as the mean ± SEM (n = 5). Bars with different superscripts differ (P < 0.05)

In the endometrium, on days 10 to 11 of pregnancy, P_4_ decreased both, OX1R gene (10, 100, 1000 nM) and protein (10, 1000 nM) expression (Fig. 3a, b). Similarly, on days 12 to 13 of pregnancy, P_4_ (10, 100, 1000 nM) caused a decrease in the receptor gene expression and protein content (Fig. 3a, b). On days 15 to 16 of gestation, P_4_ at the dose of 1000 nM increased, but at the dose of 100 nM decreased the endometrial *OX1R* gene expression. On those days, P_4_ (10, 1000 nM) suppressed the receptor protein expression (Fig. 3a, b). On days 27 to 28 of gestation, P_4_ (10 nM) decreased *OX1R* gene expression (Fig. 3a). On those days, P_4_ at the dose of 10 nM decreased, but at the doses of 100 and 1000 nM increased OX1R protein concentration (Fig. 3b). On days 10 to 11 of the oestrous cycle, P_4_ at the dose of 10 nM increased, but at the dose of 100 nM decreased *OX1R* mRNA content (Fig. 3a). On those days, P_4_ (10, 100 nM) decreased the receptor protein concentration (Fig. 3b) (P < 0.05).

**Fig. 4:**
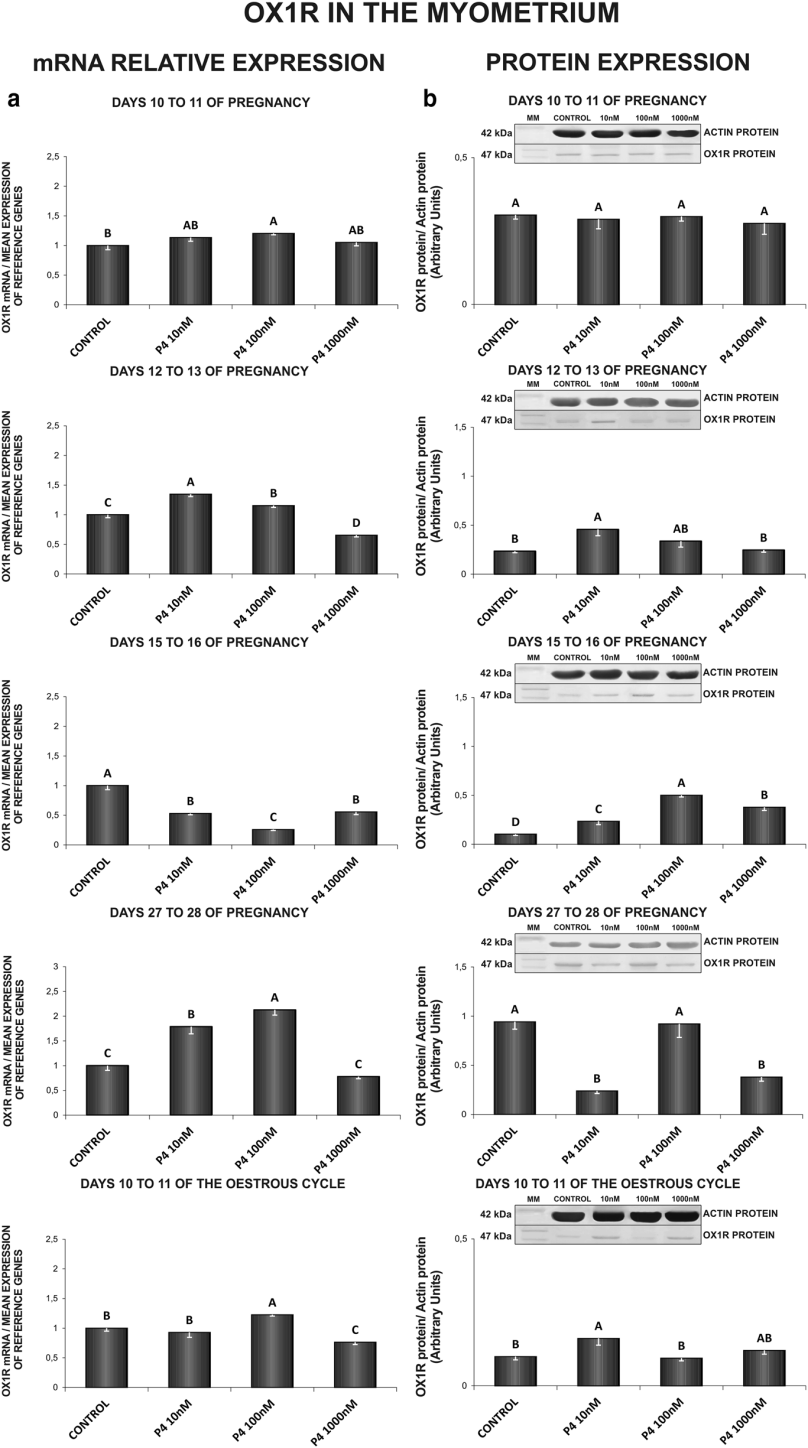
The influence of P4 (10, 100, 1000 nM) on orexin type 1 receptor (OX1R) mRNA (**a**) and protein (**b**) expression in the porcine myometrium on days 10 to 11, 12 to 13, 15 to 16 and 27 to 28 of pregnancy, and on days 10 to 11 of the oestrous cycle. The gene expression was determined by quantitative real-time PCR (**a**). The protein concentration was determined by the western blotting analysis (**b**); upper panels: representative immunoblots (MM—molecular marker); lower panels: densitometric analysis of OX1R protein relative to actin protein. Results are reported as the mean ± SEM (n = 5). Bars with different superscripts differ (P < 0.05)

In the myometrium, on days 10 to 11 of gestation, P_4_ caused an increase in *OX1R* mRNA content (100 nM) (Fig. 4a). On days 12 to 13 of pregnancy, P_4_ at the doses of 10 and 100 nM increased, but at the dose of 1000 nM decreased the receptor gene expression. On those days, P_4_ (10 nM) caused an increase in OX1R protein concentration in the myometrium (Fig. 4a, b). On days 15 to 16 of pregnancy, P_4_ inhibited *OX1R* gene expression (10, 100, 1000 nM), but enhanced the receptor protein expression (10, 100, 1000 nM) (Fig. 4a, b). Contrary, on days 27 to 28 of gestation, P_4_ increased *OX1R* mRNA content (10, 100 nM) and decreased the receptor protein expression (10, 1000 nM) (Fig. 4a, b). Progesterone, at the dose of 100 nM increased, whereas at the dose of 1000 nM decreased the myometrial *OX1R* mRNA content on days 10 to 11 of the oestrous cycle. On those days, P_4_ (10 nM) enhanced OX1R protein content (Fig. 4a, b) (P < 0.05).

### The effect of P_4_ on OX2R gene and protein expression in the endometrial and myometrial tissue explants

**Fig. 5:**
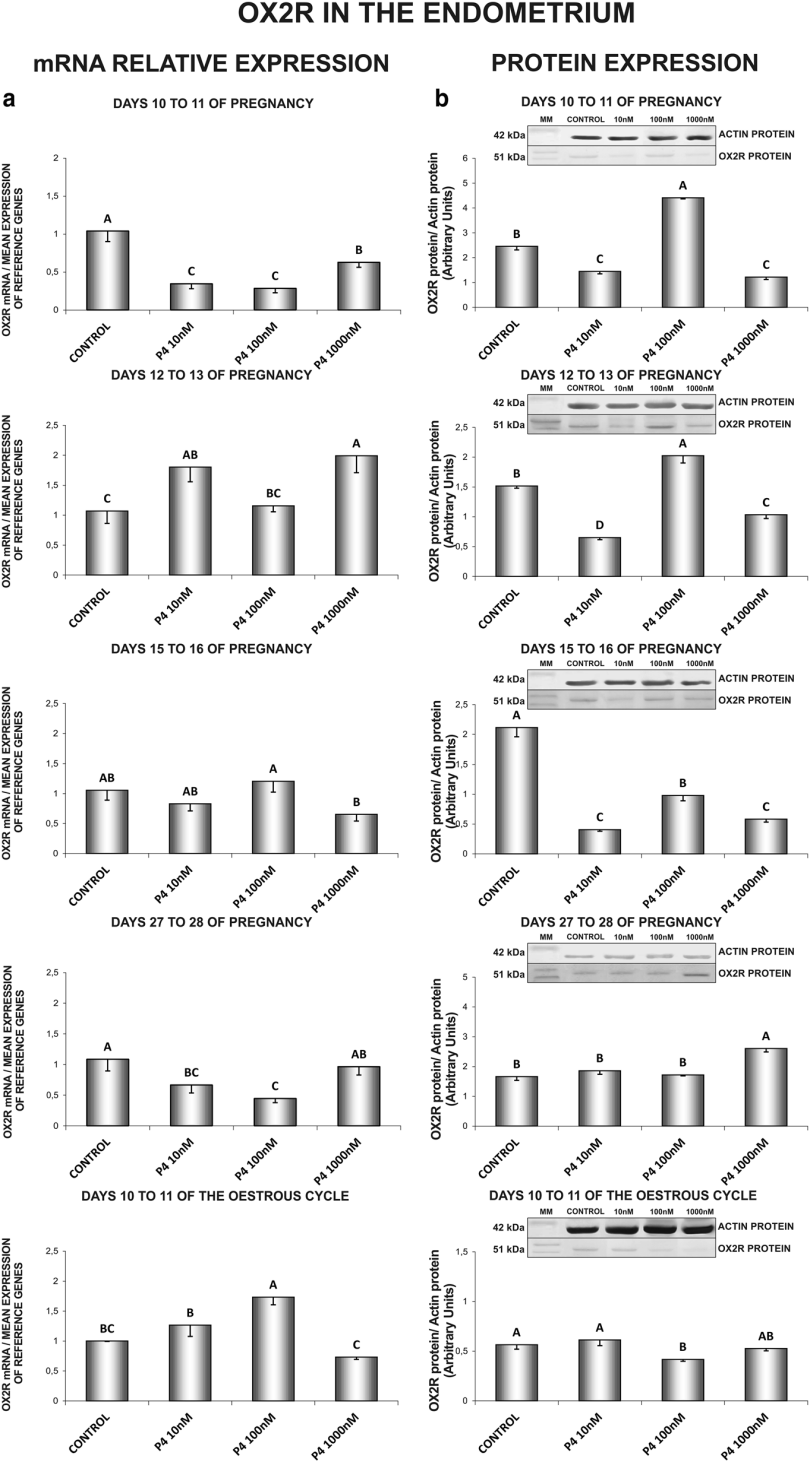
The influence of P4 (10, 100, 1000 nM) on orexin type 2 receptor (OX2R) mRNA (**a**) and protein (**b**) expression in the porcine endometrium on days 10 to 11, 12 to 13, 15 to 16 and 27 to 28 of pregnancy, and on days 10 to 11 of the oestrous cycle. The gene expression was determined by quantitative real-time PCR (**a**). The protein concentration was determined by the western blotting analysis (**b**); upper panels: representative immunoblots (MM—molecular marker); lower panels: densitometric analysis of OX2R protein relative to actin protein. Results are reported as the mean ± SEM (n = 5). Bars with different superscripts differ (P < 0.05)

In the endometrium, on days 10 to 11 of gestation, P_4_ at all tested doses caused a decrease in *OX2R* mRNA content. On those days, P_4_ at the dose of 100 nM increased OX2R protein concentration, but at the doses of 10 and 1000 nM decreased the receptor protein expression (Fig. 5a, b). On days 12 to 13 of gestation, P_4_ (10, 1000 nM) stimulated *OX2R* gene expression. On those days, P_4_ at the dose of 100 nM increased, but at the doses of 10 and 1000 nM decreased the receptor protein concentration (Fig. 5a, b). On days 15 to 16 of pregnancy, P_4_ (10, 100, 1000 nM) decreased the endometrial OX2R protein concentration (Fig. 5b). On days 27 to 28 of gestation, P_4_ suppressed the receptor gene expression (10, 100 nM), but enhanced OX2R protein concentration (1000 nM) (Fig. 5a, b). On days 10 to 11 of the oestrous cycle, P_4_ stimulated *OX2R* gene expression (100 nM) and decreased the receptor protein concentration (100 nM) (Fig. 5a, b) (P < 0.05).

**Fig. 6:**
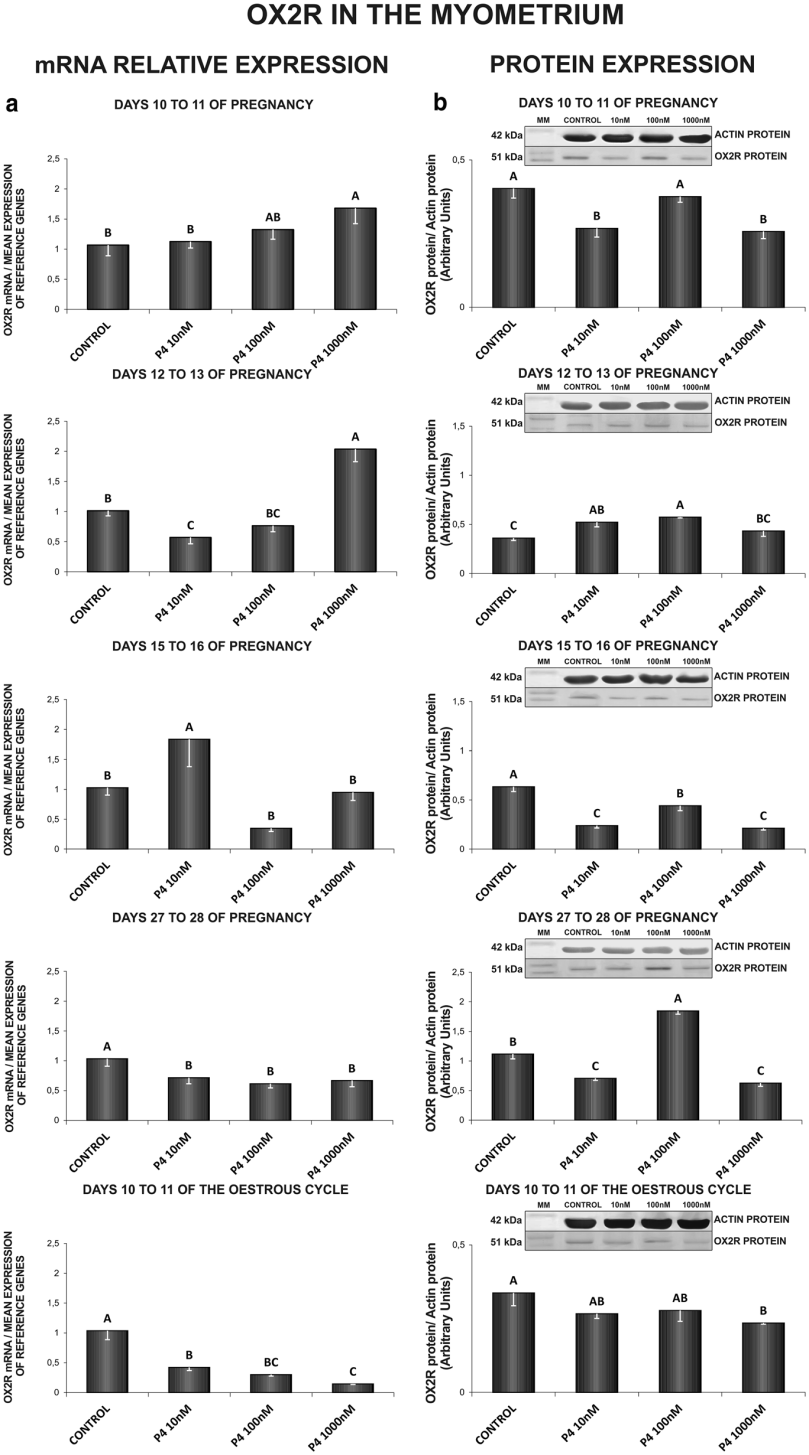
The influence of P4 (10, 100, 1000 nM) on orexin type 2 receptor (OX2R) mRNA (**a**) and protein (**b**) expression in the porcine myometrium on days 10 to 11, 12 to 13, 15 to 16 and 27 to 28 of pregnancy, and on days 10 to 11 of the oestrous cycle. The gene expression was determined by quantitative real-time PCR (**a**). The protein concentration was determined by the western blotting analysis (**b**); upper panels: representative immunoblots (MM—molecular marker); lower panels: densitometric analysis of OX2R protein relative to actin protein. Results are reported as the mean ± SEM (n = 5). Bars with different superscripts differ (P < 0.05)

In the myometrium, on days 10 to 11 of gestation, P_4_ stimulated *OX2R* gene expression (1000 nM) and suppressed the receptor protein expression (10, 1000 nM) (Fig. 6a, b). On days 12 to 13 of pregnancy, P_4_ at the dose of 1000 nM stimulated, but at the dose of 10 nM suppressed *OX2R* gene expression. On those days, P_4_ (10, 100 nM) enhanced the receptor protein concentration (Fig. 6a, b). On days 15 to 16 of pregnancy, P_4_ stimulated *OX2R* gene expression (10 nM) and inhibited the receptor protein content (10, 100, 1000 nM) (Fig. 6a, b). On days 27 to 28 of pregnancy, P_4_ at all tested doses inhibited the receptor gene expression. On those days, P_4_ at the dose of 100 nM stimulated, but at the doses of 10 and 1000 nM inhibited the myometrial OX2R protein expression (Fig. 6a, b). On days 10 to 11 of the oestrous cycle, P_4_ diminished on both, *OX2R* gene (10, 100, 1000 nM) and protein (1000 nM) expression (Fig. 6a, b) (P < 0.05).

## Discussion

The expression of the *PPO* gene, OXA and OXB proteins and OX1R and OX2R genes and proteins in the uterus during the oestrous cycle and early pregnancy has been previously reported [13, 14]. The orexin system has been localised in the porcine uterus, however, there is a general scarcity of data describing the relationship between orexins and the factors regulating reproductive function, including steroid hormones and prostaglandins. The results of this study indicate that P_4_ has a modulatory effect on the expression of the orexin system (orexins and their receptors) during early gestation. The modulatory effects of P_4_ were observed at both gene and protein expression level. In the present study, the influence of P_4_ on orexins and their receptors was different in each stage of gestation, when compared to the controls. This differences may be caused by differential expression of P_4_ receptors during the examined periods. What is more, during early pregnancy period uterine tissues undergo an intensive remodelling process to prepare the uterus for the implantation, and placentation [24], what results in different cell populations and, in consequence, different tissues receptivity for P_4_. The observed effect was also tissue- and dose-dependent. Our study revealed different expression patterns of PPO, OX1R and OX2R genes and proteins, in relation the control samples. The different expression patterns of mRNA and proteins is not surprising. In mammals, the correlation between genes and proteins expression can be as low as 40%, and it is influenced by many factors, including mRNA and protein stability, transcriptional and post-transcriptional regulation, functioning feedbacks that suppress gene expression through high protein concentrations, and attenuation of post-transcriptional processes through high mRNA content [25, 26].

In mammals, the lifespan of the corpus luteum (CL) has to be extended for continued P_4_ production and the establishment of a successful pregnancy. The maintenance of P_4_ secretion is pivotal for the development of the conceptus and implantation [25]. Oestrogens are produced by porcine conceptuses on around day 11 of pregnancy, they initiate changes in the secretion of the main luteolytic factor, prostaglandin F_2α_ (PGF_2α_), from endocrine to exocrine secretion, and increase the production of prostaglandin E_2_ (PGE_2_), a luteoprotective mediator [15, 27]. The main aim of the oestrogen-induced decrease in PGF_2α_ concentration in uterine venous blood is to protect CL, maintain uninterrupted synthesis of P_4_ and, consequently, to guarantee the proper course of gestation [15]. The ovaries are the main source of P_4_ in the body, but the hormone may be also synthesized by uterine tissues. Our previous study demonstrated that despite the decrease in the blood plasma concentration of P_4_ during early pregnancy, P_4_ levels in uterine luminal fluid remained constant between days 12 to 28 of gestation and were even higher in comparison with days 10 to 11 of pregnancy [18]. The steroidogenic activity of the porcine endometrium and myometrium was reported by Franczak [19]. Wojciechowicz et al. [28] and Smolinska et al. [20] observed the expression of 3β-hydroxysteroid dehydrogenase/Δ5–Δ4 isomerase and P_4_ secretion by endometrial and myometrial tissues during both early pregnancy and the oestrous cycle. For more, Wojciechowicz et al. [18] indicated, that during early pregnancy the uterine tissues release daily about 2000 pg of P_4_ per gram of uterus. The above suggest that uterine tissues may play an important role as an alternative source of P_4_.

Orexins are the physiological mediators of food intake and appetite [2]. Fasting and insulin-induced hypoglycaemia have been found to increase *PPO* mRNA in the rat lateral hypothalamic area [2, 29]. Lu et al. [30] also demonstrated that fasting regulates the hypothalamic expression of orexin receptors. Most research investigating the role of orexins in food intake focuses on the central nervous system. Generally, an increase in orexin concentrations in the brain stimulates appetite and food deprivation results in an increased expression of the orexin system in the brain [30–33]. Karteris et al. [34] observed considerable differences between orexin receptors in the adrenal cortex and the hypothalamus of rats. In the hypothalamus, food deprivation induced OX1R and OX2R expression at both gene and protein level, whereas in the adrenal glands, OX1R and OX2R concentrations were significantly reduced in food-deprived animals. Based on the above findings, we hypothesized that a similar response may take place in the uterus when rapid embryonic development leads to energy depletion. In the present study, P_4_ inhibited the expression of OX1R protein in nearly all analyzed periods, whereas the expression of OX2R protein was inhibited only on days 15 to 16 of gestation. Our results suggest that P_4_ could influence orexins indirectly by modulating the expression of their receptors. Our previous study revealed that orexins could affect steroidogenesis in the porcine uterus. Both OXA and OXB modulated the expression of genes encoding key steroidogenic enzymes, cytochrome P450c17 and cytochrome P450 aromatase, in endometrial and myometrial tissues. OXA and OXB also regulated the secretion of oestrone, oestradiol and testosterone by uterine tissues in a dose-dependent manner. The influence of both orexins differed between the investigated periods of gestation, which could be attributed to changes in the concentrations of OX1R and OX2R and the OX1R:OX2R ratio [35, 36]. Progesterone could also be involved in the autoregulation of steroidogenesis by controlling orexin receptivity in the uterus.

Gestation is a critical period for both the mother and the foetus, and its proper course depends on a number of maternal factors. The maternal organism supplies nutrients such as glucose, amino acids and lipids to the developing embryo via the placenta [37]. In mammals, numerous metabolic changes, such as adaptation of carbohydrate metabolism, occur during pregnancy. During gestation, progressive insulin resistance inhibits maternal glucose utilization and increases glucose transport to the foetus. Low glucose levels may increase the risk of miscarriage by depriving the placenta and the foetus of the principal substrate for growth [38–40]. Progressive insulin resistance during normal gestation is associated with an increase in maternal P_4_ serum levels. Orexins exert similar effects on insulin and carbohydrate metabolism. Nowak et al. [41] observed that both orexins stimulate insulin secretion by rat pancreatic cells, both in vivo and in vitro. In the current study, P_4_ had a minor stimulating effect on OXA (on days 10 to 11 of gestation) and OXB (on days 12 to 13 of gestation) secretion by the endometrium. It is also possible that orexin levels increase during embryo migration and early recognition of pregnancy to prepare the endometrium for implantation. However, the P_4_-mediated decrease in the concentrations of both orexin receptors during implantation could also desensitise the endometrium to orexins and promote insulin resistance, characteristic for normal gestation.

Orexins participate in the regulation of reproductive function by influencing the hypothalamic-pituitary-ovarian axis. Our previous findings [13, 14] indicate that the orexin system is expressed in the porcine uterus and is dependent on the local hormonal milieu. Progesterone could be responsible for the local regulation of orexin activity during pregnancy. In another study (Smolinska et al., data unpublished), we demonstrated that other uterine factors, including prostaglandins (E_2_ and F_2α_), oestrone and oestradiol, could participate in the regulation of orexin activity in the uterus. Moreover, we observed that orexins could exert modulatory effects on steroidogenesis in the porcine uterus [35, 36]. This points to the presence of a local feedback loop between orexins and steroids in a pregnant uterus that could play an important role in the regulation of maternal metabolism during pregnancy. However, further research is needed to validate the above hypothesis.

## Conclusions

The modulatory effects of P_4_ on the orexin system in a pregnant uterus is reported for the first time. The presented data may contribute to the existing knowledge of the mechanisms linking maternal energy metabolism with the regulation of the reproductive system during pregnancy. The results of this study expand our knowledge of the processes that take place in a pregnant uterus and the role, which P_4_ and orexins play in the uterus during gestation.
